# Highly dynamic temporal changes of TSPY gene copy number in aging bulls

**DOI:** 10.1371/journal.pone.0178558

**Published:** 2017-05-26

**Authors:** Olutobi A. Oluwole, Kiana Mahboubi, Laura A. Favetta, Tamas Revay, Tom Kroetsch, William Allan King

**Affiliations:** 1 Department of Biomedical Sciences, University of Guelph, Guelph, ON, Canada; 2 Semex, Guelph, ON, Canada; Embrapa, BRAZIL

## Abstract

The Y-chromosomal TSPY gene is one of the highest copy number mammalian protein coding gene and represents a unique biological model to study various aspects of genomic copy number variations. This study investigated the age-related copy number variability of the bovine TSPY gene, a new and unstudied aspect of the biology of TSPY that has been shown to vary among cattle breeds, individual bulls and somatic tissues. The subjects of this prospective 30-month long study were 25 Holstein bulls, sampled every six months. Real-time quantitative PCR was used to determine the relative TSPY copy number (rTSPY CN) and telomere length in the DNA samples extracted from blood. Twenty bulls showed an altered rTSPY CN after 30 months, although only 9 bulls showed a significant change (4 significant increase while 5 significant decrease, *P*<0.01). The sequential sampling provided the flow of rTSPY CN over six observations in 30 months and wide-spread variation of rTSPY CN was detected. Although a clear trend of the direction of change was not identifiable, the highly dynamic changes of individual rTSPY CN in aging bulls were observed here for the first time. In summary we have observed a highly variable rTSPY CN in bulls over a short period of time. Our results suggest the importance of further long term studies of the dynamics of rTSPY CN variablility.

## Introduction

While copy number variations (CNVs) occur throughout the mammalian genome, the Y-chromosome is particularly interesting. Its’ appeal lies in the fact that its’ structure is rich in multicopy genes and repeats that are prone to be involved in intra-chromosomal rearrangements, thus frequently leading to CNVs [1]. Although, genetic variation of many Y-chromosomal genes has been associated with non-reproduction related phenotypes, such as autoimmune conditions, heart disease or cancer, most of the highly ampliconic gene families show male specific expression patterns and play specific roles in reproduction [2]. Testis specific protein, Y encoded (TSPY) is one such multicopy Y chromosomal gene expressing in the testis with a suggested function in spermatogenesis [3]. In many mammalian species, it is arranged in tandemly repeated clusters that facilitates the extensive variation of the described otherwise high copy number [4]. In humans TSPY CN varies between 11 and 76 [5] that is surpassed by the detected CN range of 50–200 copies in cattle [6]. Multicopy status was described for other mammals, like primates, dogs, horses and cats, however its CN variability was not always investigated in these species [3,4]. Interestingly, pigs have three copies that do not show variation [7], rats have only one copy and no functional TSPY copy is present in mice [8].

TSPY is among the most studied Y-chromosomal gene in cattle and extensive copy number variation was described among 14 different breeds, as well among individual bulls of the same breed [6]. Further, significant differences in TSPY CN were recently found among somatic tissues of individual bulls [9]. Higher CN were detected in tissue types with higher cell division rate (e.g. blood and testis) while those with lower cellular turnover (e.g. brain and muscle) had relatively lower CN as well. This is in agreement with the suggested role of somatic divisions specifically mitotic recombination and DNA repair mechanisms in generating mosaic CNV events [10]. Progressive loss of the integrity of fundamental cellular functions, such as the precise control of cell divisions is a hallmark of aging, as it is observed by the increase of genomic instability over time. Additionally, the inactivity of the DNA repair mechanisms to re-build chromosome ends in most adult cell types, leads to the well-known gradual attrition of the telomeres, considered as a marker of cellular aging [11].

We hypothesize that an individual’s TSPY CN changes over time, just as the burden of common autosomal CNVs was observed in large studies of aging populations [12]. This study investigates the age-related copy number variability of the bovine TSPY gene, a new and unstudied aspect of the biology of TSPY that has been shown to vary among cattle breeds, individual bulls and somatic tissues. Additionally, we investigate the change in telomere length, as a benchmark for aging.

## Materials and methods

### Sample collection and DNA extraction

The subjects of this prospective 30-month long study were 25 Holstein bulls of similar age maintained in the same artificial insemination centre under the same housing and feeding conditions. At the outset, these animals were young bulls entering an AI program and were not selected for research purposes. Blood samples from fifteen animals were sampled every 6 months providing 6 observations (T0, T6, T12, T18, T24, T30) and the additional ten bulls were collected at the beginning and end of the study (2 time points per animals, T0 and T30). Sampling was done by veterinarians as part of routine animal health check and mandatory sampling for CFIA (Canadian Food Inspection Agency) testing, according to the Canadian Council on Animal Care and University of Guelph’s Animal Care Committee guidelines. DNA was extracted from blood samples using the standard phenol chloroform extraction and precipitation method as previously described [6].

### Determination of TSPY copy number

Real-time quantitative PCR (qPCR) was used to determine the relative TSPY copy number (rTSPY CN) in the samples. rTSPY CN was calculated after normalization against the single copy SRY and a Calibrator sample according to the efficiency corrected ΔΔC_T_ method [13]. In order to reflect the temporary changes the normalized values were compared relative to the corresponding T0 of the given animal, thus named as rTSPY CN. DNA specific primers for TSPY and SRY were described earlier [6,9] and listed in S1 Table. The 10ul reaction containing 4.5mM primers, 6ng of genomic DNA and 1X SsoFast EvaGreen Supermix (Bio-Rad) was carried out in a CFX96 Touch™ Real-time PCR Detection System (Bio-Rad) with denaturation at 98^°^C for 2 min, followed by 50 cycles of 98^°^C for 5s, 60^°^C for 5s and 72^°^C for 10s. Melting curve was obtained between 72^°^C and 95^°^C with measurements taken every 0.2^°^C. All samples were run in triplicates. Primer efficiency was calculated from standard curves created for both sets of primers according to the equation E = 10^−1 / slope^. The rTSPY values were tested for normality with D’Agostino and Pearson, Shapiro-Wilk and Kolgomorov-Smirnov tests, then Student’s t-test was used to for statistical comparisons.

### Determination of relative telomere length

Relative telomere length were determined by the standard qPCR protocol against the single copy reference gene ZAR-1 and the Calibrator [14,15]. qPCR was carried out in a CFX96 Touch™ Real Time–PCR Detection System (Bio-Rad) in 10ul reaction containing 6ng of genomic DNA, 1X SsoFast EvaGreen Supermix (Bio-Rad), and 5mM of primers (S1 Table). Samples were run in triplicates. Student’s t test was used to determine the difference in relative telomere length in each animal between the start and end time points of the study (T0 and T30). A *P* value of < 0.01 was used to establish statistical significance.

## Results

rTSPY CN was determined for all bulls at T0 and revealed extensive variation among the individual bulls (S1 Fig). Seventeen out of 25 bulls showed significantly different TSPY copy number when compared to the calibrator sample. Fifteen animals had significantly higher and two animals had significantly lower rTSPY CN at the beginning of the 30-month experiment.

**Fig 1:**
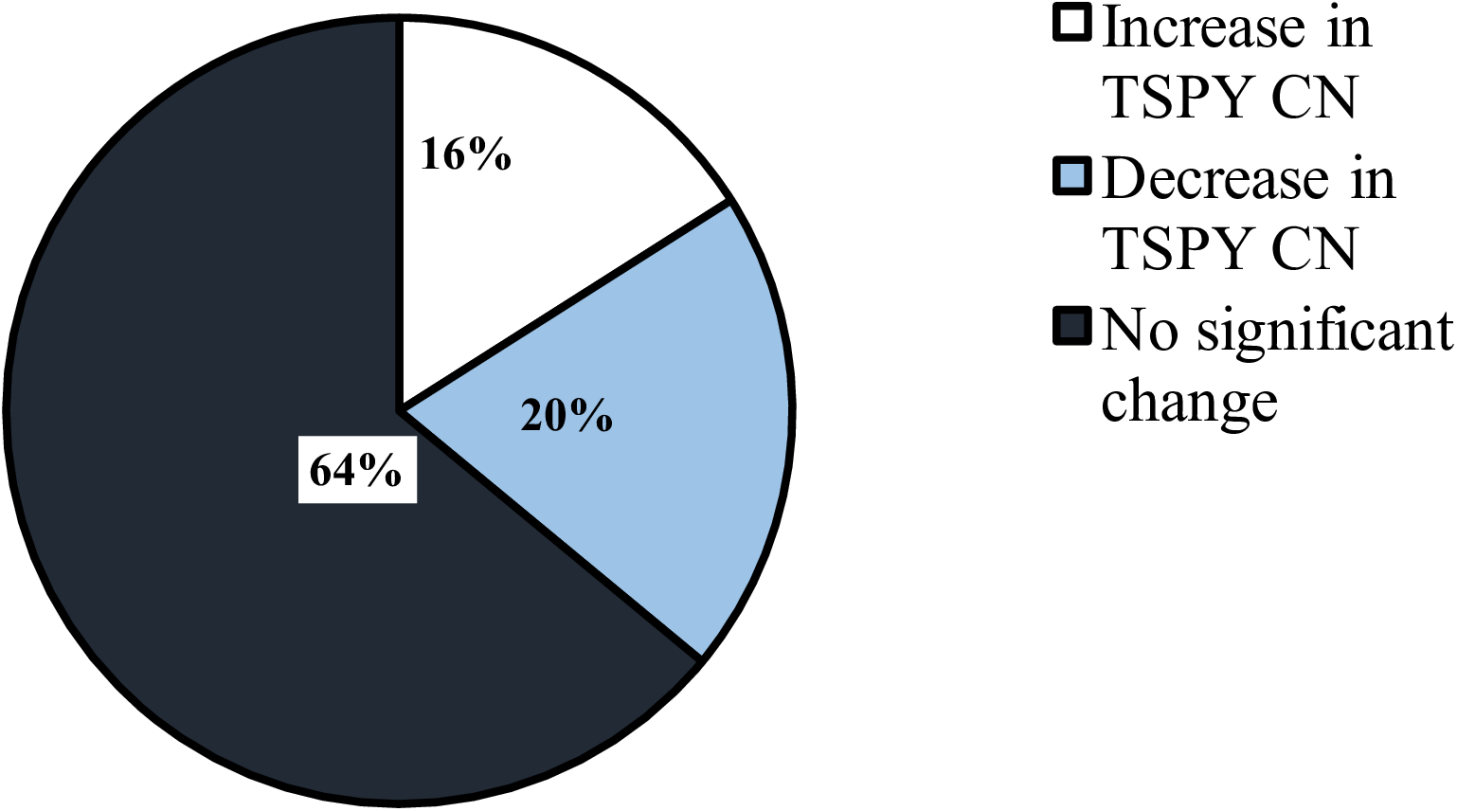
Pie chart representation of change in TSPY copy number in the 25 bulls over a period of 30 months relative to 0-month samples. P≤0.01.

**Fig 2:**
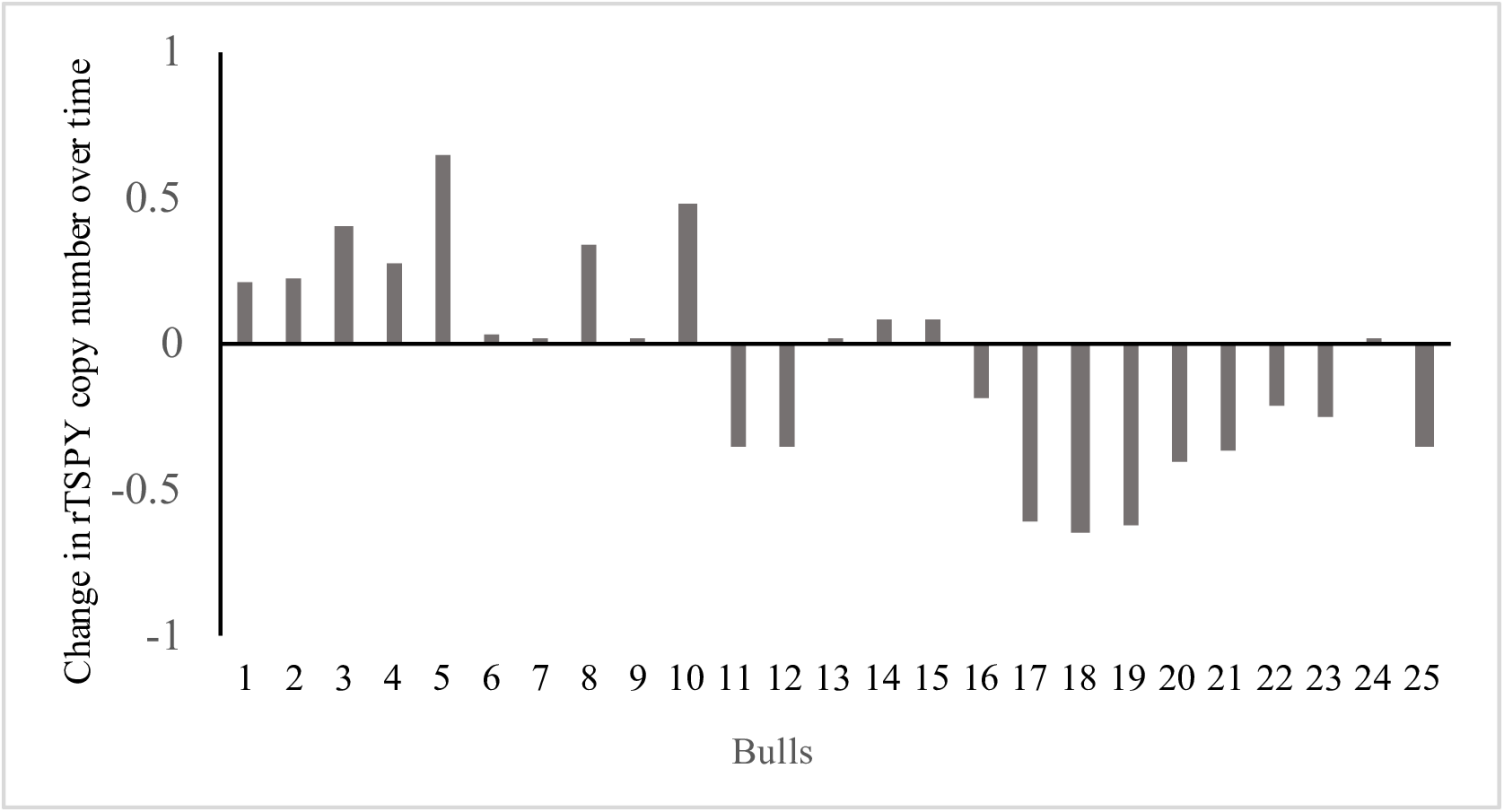
Relative TSPY copy number change over the 30 months sampling period (rTSPY CN at T30—rTSPY CN at T0) in each individual bull.

The overall change in rTSPY CN during the 30-month sampling period was calculated for all 25 bulls by comparing T30 vs. T0 (Figs 1 and 2). Twenty bulls showed an altered rTSPY CN after 30 months, although only 9 bulls (36%) showed a significant change (*P*<0.01). Of those 9 bulls, 4 (44.4%) showed a significant increase (*P*<0.01) while 5 (55.6%) showed a significant decrease (*P*<0.01) in rTSPY CN over time. Out of the 16 remaining animals, rTSPY CN of 5 bulls did not change at all in 30 months when the two end-point observations (T30-T0) are compared.

**Fig 3:**
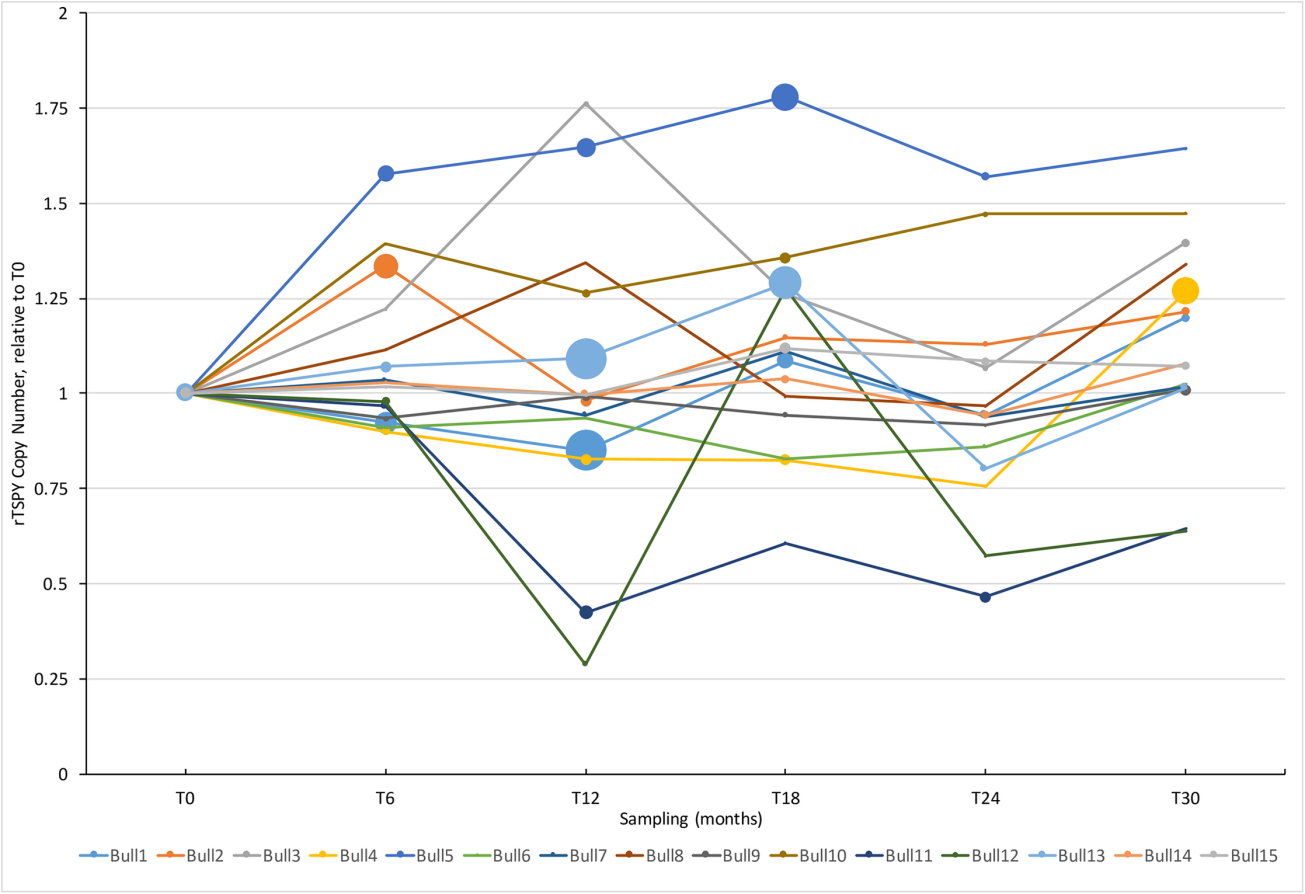
rTSPY CN measured by qPCR at every six months (T0-T30) during the 30 months experiment. The rTSPY CN is calculated against T0 (thus CN = 1). A line diagram representing all 15 bulls, The size of the circles at each time points are proportional to rTSPY CN±SEM.

To get a detailed view of rTSPY CN variability over time, qPCR was performed on samples from 15 bulls for all six sampling time point over the 30-month period. Analysis of the rTSPY CN of these bulls revealed a highly variable rTSPY CN that nearly doubled the starting copy number (at T0) in some animals. Most animals showed an increase or decrease of rTSPY CN within six months (two consecutive observations) and 11 changed significantly. A clear trend in the direction of rTSPY CN change was not identifiable (Fig 3).

The relative telomere length was calculated for fifteen bulls at T0 to reveal inter-individual variation at the beginning of the sampling period. The telomere length varied significantly (*P*<0.01) in 11 bulls when compared with the calibrator (S2 Fig).

We also determined change in relative telomere length (RTL) over time and we observed minimal variability between the start and end points (T30 vs T0). Thirteen bulls (87%) displayed no significant difference in RTL, while 2 bulls showed a slight, but significant increase (P<0.01). One of these two bulls showed an increased rTSPY CN (T30-T0), while the other did not.

## Discussion

The TSPY gene is situated on the mammalian Y-chromosome and represents a unique biological model to study various aspects of genomic copy number variations, such as its dynamics in aging. Not only TSPY is one of the highest copy number protein coding gene in the mammalian genome, but its extensive individual variation was also observed [3]. The subjects of this study were 25 Holstein bulls that were tested for their variability in rTSPY CN at the beginning of the 30-month sampling period. Most animals (17 of 25) had significantly different copy number at T0 when compared to a common calibrator sample (S1 Fig). It is consistent with the observations of Hamilton et al [6] who found significant variability among Holstein bulls and also among several cattle breeds. Similarly, crossbred Bos taurus x Bos indicus bulls showed extensive individual rTSPY CN variations [16]. This extensive structural plasticity is thought to occur due to the genomic organization of TSPY copies into an amplified block that provides ample opportunities for intra chromosomal rearrangements leading to the change of copy numbers. The bovine TSPY locus consists of up to hundreds of highly similar copies in a tandem arranged structure, as revealed by the sequence and FISH map of the Y-chromosome [6,17]. These repeats can mislead the otherwise highly precise mechanisms of DNA recombination and repair due to the pairing of repeat units from different positions along the chromosome and among the two sister-chromatids. Non-allelic homologous recombination (NAHR), unequal sister-chromatid exchange (USCE) and various other DNA strand break repair mechanisms rely on potential faulty recognition among many homologous sequences and thus play key roles in generating CNVs [18]. This is emphasized by the contrasting stability of TSPY CN in pigs, where only three copies of the gene are available for recombination [7]. The observed and above discussed individual variability of rTSPY CN could be explained by the action of these recombination mechanisms during meiosis, as the resulting embryo and developing bull would carry an altered TSPY locus. Furthermore, the role of somatic divisions and DNA break repair events in generating CNVs should also be considered, as emphasized by recent findings of extensive rTSPY CN variability among somatic tissues in bulls [9]. There, differential rates of somatic mosaicism were shown between fast and slowly regenerating tissues, by detecting greater CNVs in blood and testis vs. brain or muscle. This logically implies that the number of divisions, thus the elapsed time and aging could affect the CNV landscape of an individual. Although only limited information is available, studies of aging human populations showed an increasing burden of CNVs [19], as well as associations of specific CNVs with mortality and lifespan [12]. Investigation of the dynamics of a Y-chromosomal ampliconic gene, such as TSPY during aging is not available in the literature. Thus, our main objective was to detect the rTSPY CN variation over time in a prospective design by sampling individual bulls in every six months for almost three years. For reliable comparison among all samples, we have calculated the relative copy numbers of TSPY against the first sample collected from the same bull by qPCR. This provided the flow of rTSPY CN over six observations in 30 months (Fig 3). We have detected wide-spread variation of rTSPY CN in every six months. Although a clear trend of the direction of change was not identifiable, the highly dynamic changes of rTSPY CN in aging bulls were observed here for the first time.

Although positive selection pressure for evolutionary CN amplification of TSPY in various species suggests male-specific function, its exact cellular role is still mainly unknown [3,20]. TSPY expression is specific to the testis and its protein product was localized in spermatogonia within seminiferous tubules [21]. Its conserved domain structure and interaction with cyclins suggest functions in the regulation of the testicular cell cycle and as a factor involved in the proliferation of testicular germ cells [21,22]. Studies in bulls have shown that TSPY copy number correlates with fertility [6,23] and several semen quality parameters [16]. Long term aging itself has an adverse effect on semen volume, sperm motility and morphology as evidenced from the numerous reports in men [24,25] and bulls [26]. Here we sampled AI bulls for 30 months, thus these animals have not yet reached the age where the decline of semen quality could be expected. This was also reflected by the detected relative telomere length data that did not change significantly in all but two animals. Although telomere attrition is a general hallmark of aging, recent studies defined this phenomenon as a long term marker and high individual variability was observed in shorter periods, just as during the 30 months covered by this study [11,27,28]. This variation includes the detectable lengthening of telomeres, that is most probably due to the mosaic nature of lymphocites and potenial loss of cells with shorter telomeres [11].

In summary we have observed a highly variable rTSPY CN in bulls over a short period of time. Our results suggest the importance of further long term studies of the dynamics of rTSPY CN variablility and its potential association with prolongation of male fertility.

## Supporting information

S1 Fig Relative TSPY (rTSPY) copy number of 25 bulls at first sample collection (0- month). *marks samples with significantly different rTSPY CN as compared to the calibrator sample (CALI) *P*<0.01.
(TIF)

S2 Fig Relative telomere length in bulls at first sample collection (0 month). *P*<0.01.
(TIF)

S1 Table Primer sequences used for real time PCR experiments. (XLSX)
