# Supplementary figures and images for: Highly dynamic temporal changes of TSPY gene copy number in aging bulls
Source: PLoS One. 2017 May 26;12(5):e0178558. doi: 10.1371/journal.pone.0178558 (PMC5446161; doi:10.1371/journal.pone.0178558)

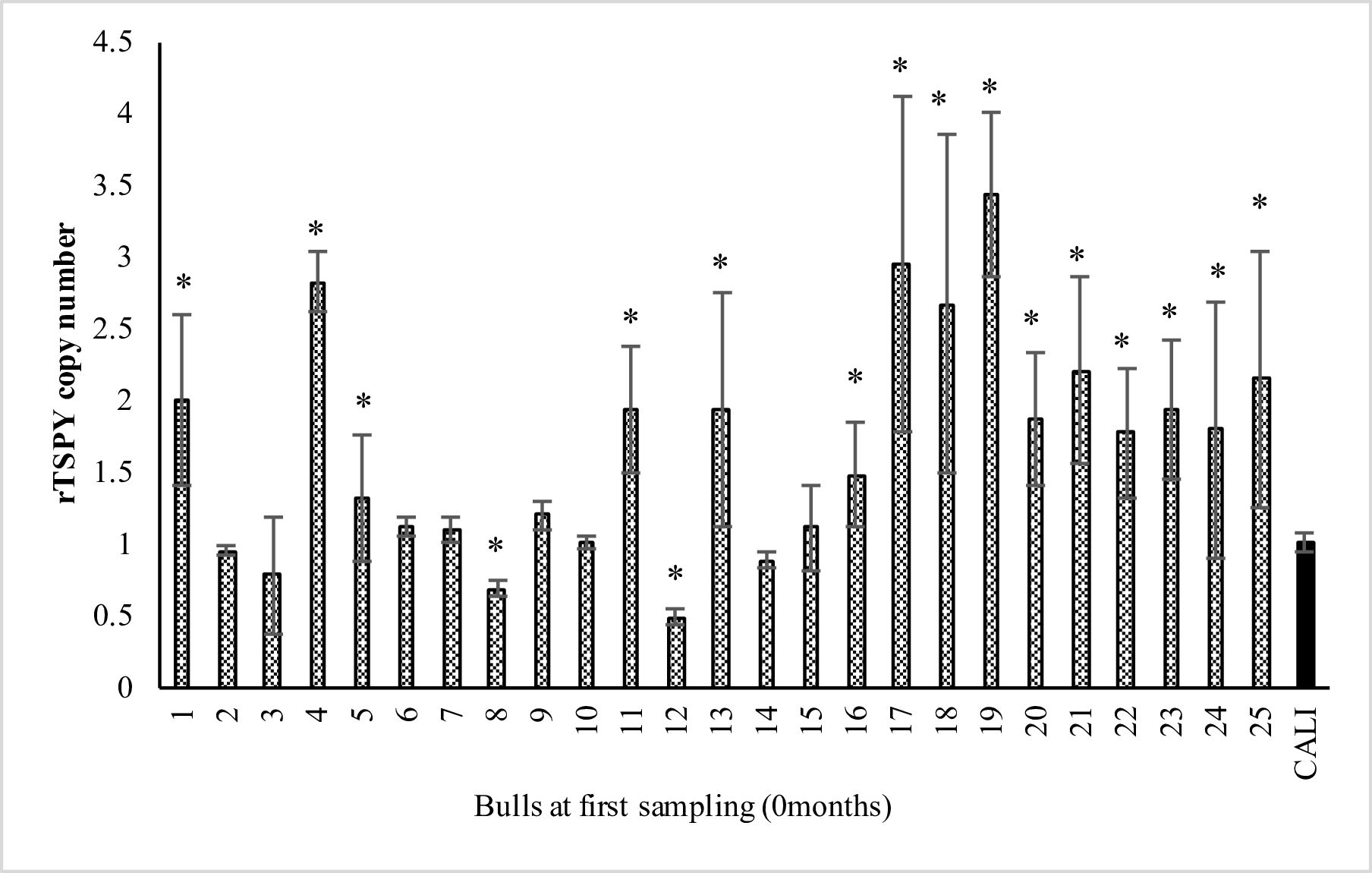

Supplement: S1 Fig — *marks samples with significantly different rTSPY CN as compared to the calibrator sample (CALI) P<0.01. (TIF) [file pone.0178558.s001.tif]

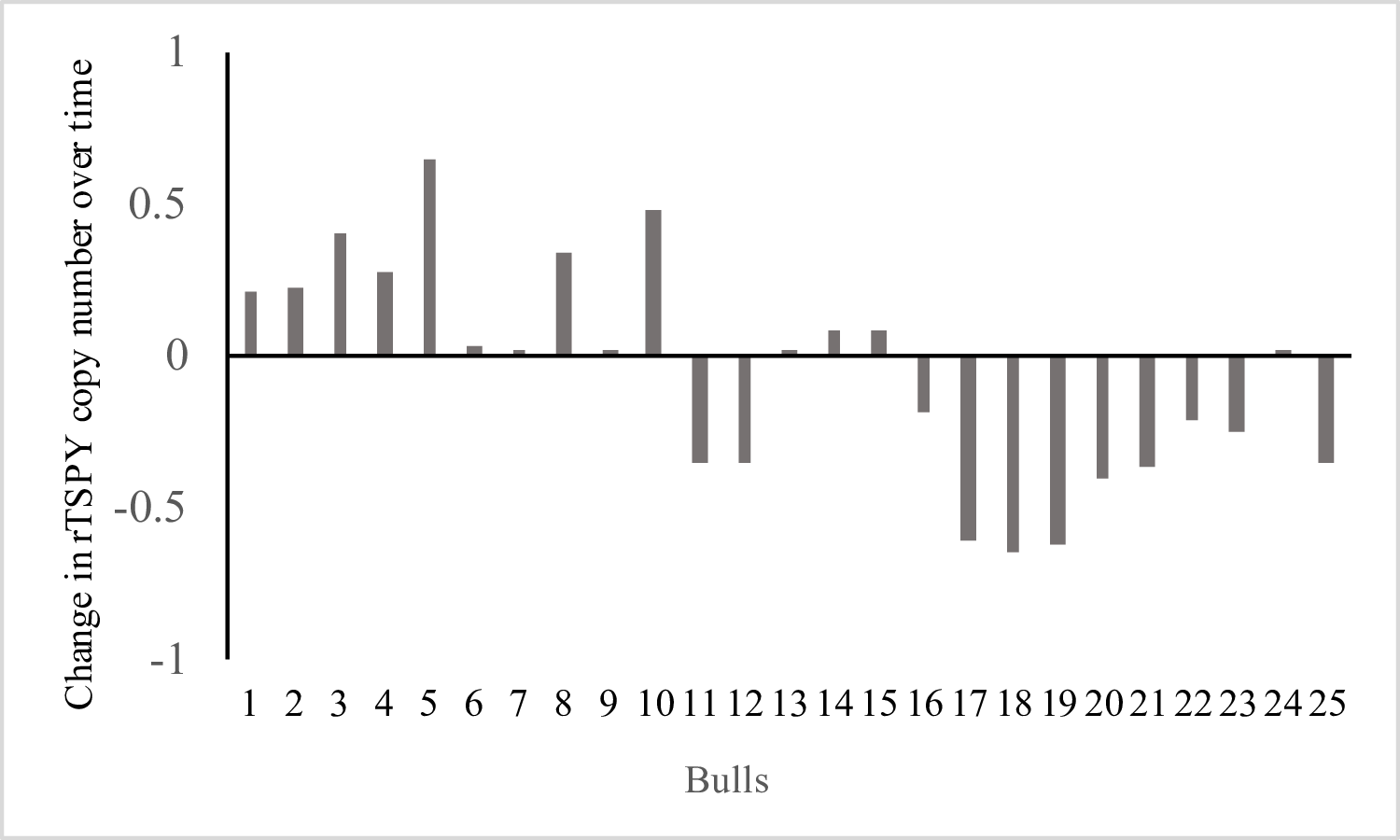

Supplement: S2 Fig — P<0.01. (TIF) [file pone.0178558.s002.tif]
